# Demonstration of the potential of white-box machine learning approaches to gain insights from cardiovascular disease electrocardiograms

**DOI:** 10.1371/journal.pone.0243615

**Published:** 2020-12-17

**Authors:** Thilo Rieg, Janek Frick, Hermann Baumgartl, Ricardo Buettner

**Affiliations:** Machine Learning Research Group, Aalen University, Aalen, Germany; Texas A&M University, UNITED STATES

## Abstract

We present the results from a white-box machine learning approach to detect cardiac arrhythmias using electrocardiographic data. A C5.0 is trained to recognize four classes using common features. The four classes are (i) atrial fibrillation and atrial flutter, (ii) tachycardias (iii), sinus bradycardia and (iv) sinus rhythm. Data from 10,646 subjects, 83% of whom have at least one arrhythmia and 17% of whom exhibit a normal sinus rhythm, are used. The C5.0 is trained using 10-fold cross-validation and is able to achieve a balanced accuracy of 95.35%. By using the white-box machine learning approach, a clear and comprehensible tree structure can be revealed, which has selected the 5 most important features from a total of 24 features. These 5 features are ventricular rate, RR-Interval variation, atrial rate, age and difference between longest and shortest RR-Interval. The combination of ventricular rate, RR-Interval variation and atrial rate is especially relevant to achieve classification accuracy, which can be disclosed through the tree. The tree assigns unique values to distinguish the classes. These findings could be applied in medicine in the future. It can be shown that a white-box machine learning approach can reveal granular structures, thus confirming known linear relationships and also revealing nonlinear relationships. To highlight the strength of the C5.0 with respect to this structural revelation, the results of further white-box machine learning and black-box machine learning algorithms are presented.

## Introduction

The prediction of machine learning (ML) algorithms has achieved great progress in the detection of diseases [1–4]. This has mostly been enabled by using algorithms with deep structure. However, such black-box ML approaches do not cover the area of cause-effect relationships in detail. How exactly the results are achieved is difficult to understand [5]. For this reason, it makes sense to use white-box ML approaches. The decision structure can be understood with these approaches, which is why they are very well suited for application in the medical field [6]. White-box ML approaches can be used to confirm known linear relationships and to discover new nonlinear relationships and interpret the results in a more granular way. Such linkages can contribute important knowledge for medicine, where decisions are associated with high risks. A wrong decision in medicine can have serious consequences for the patient; therefore, high transparency and interpretability in the decision making process are important [6]. White-box ML approaches could consequently play a key role in medicine in the future [7].

**Fig 1:**
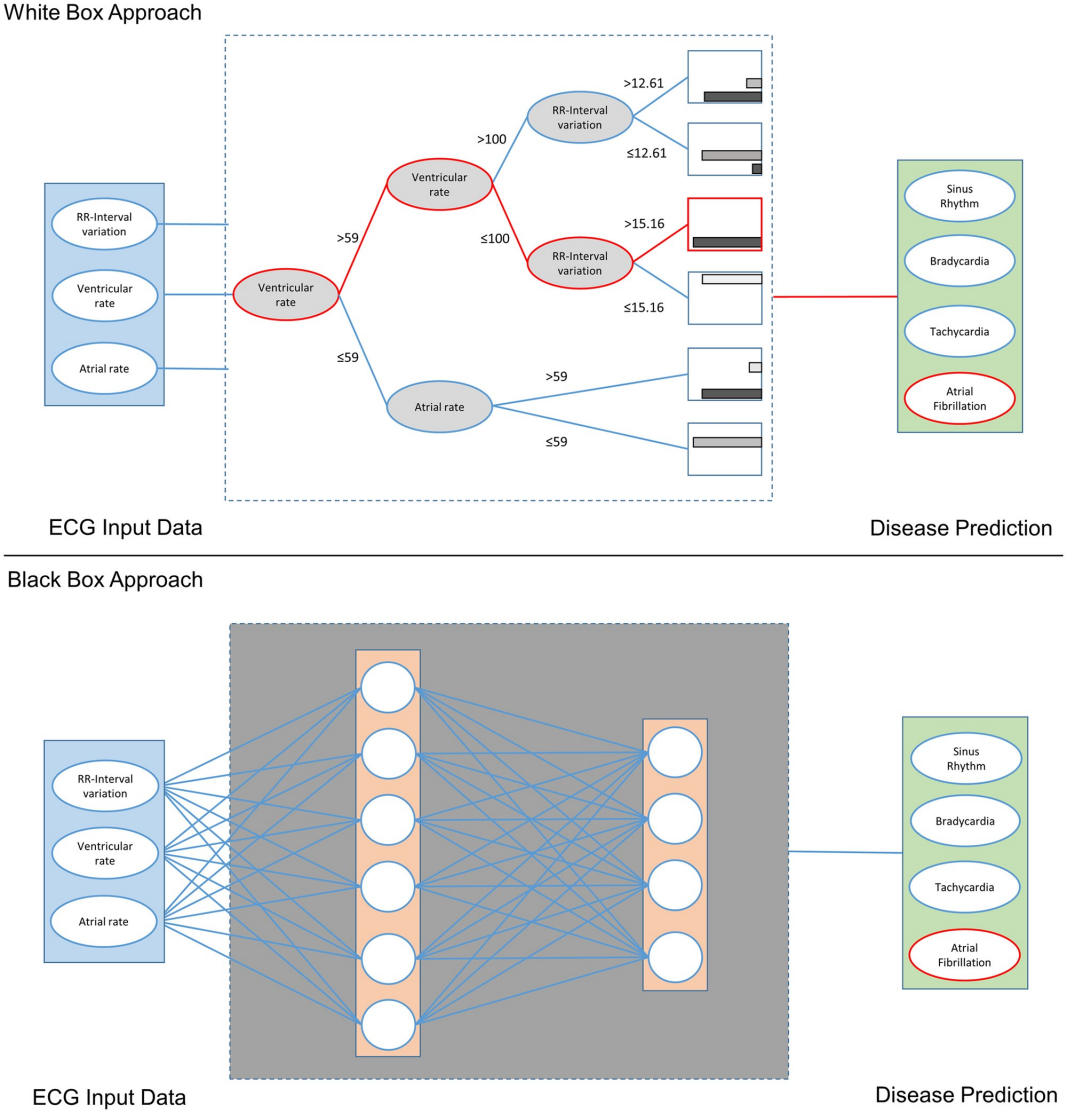
Schematic comparison of white-box and black-box ML approaches.

As shown in Fig 1, each ML model uses input data (blue) to train a predictive ML model that provides a prediction (green). Black-box ML approaches are difficult or impossible to understand. On the contrary, white-box ML approaches are characterized by the fact that the structure with which an algorithm produces a result can be revealed, and therefore, they can easily be understood by experts in the field [8]. One ML algorithm that is particularly suitable for this purpose is the C5.0, which exhibits very good performance and explains in detail how it achieved the result [9].

In the field of cardiovascular diseases in particular, such white-box ML approaches can lead to considerable improvements. Cardiovascular disease is the leading cause of mortality worldwide. These diseases are responsible for 30% of deaths [10]. Half of these deaths are caused by cardiac arrhythmias, which represent an entire family of diseases. Arrhythmias can be detected in patient electrocardiograms (ECG). Each ECG is evaluated by cardiologists or other physicians. This evaluation by humans is still prone to errors: on average, cardiologists reach an F1 value of 0.780 [1]. Therefore, the evaluation is almost always supported by commercial computer software. However, various studies indicate substantial misdiagnosis [11, 12]. To minimize this error rate, it is important to further explore the signals of the ECG. Features and nonlinear relationships must be found that can be taken into account in human and software diagnosis.

**Fig 2:**
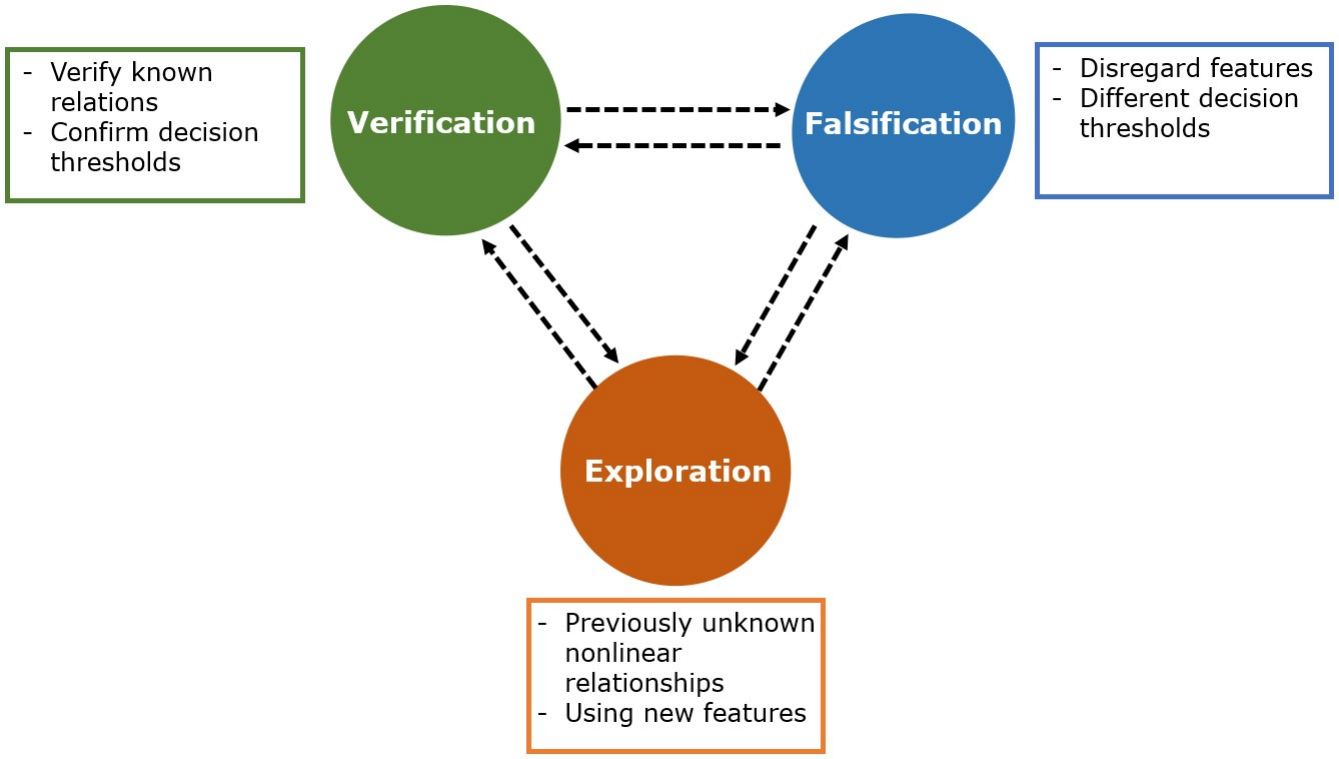
C5.0 in the scope of scientific theory.

Besides the practical implications of the white-box ML approaches, they can also significantly contribute to the scientific discourse. The algorithm as a neutral, statistically based tool that makes decisions without bias can be used for scientific verification, falsification and exploration (see Fig 2). The algorithms offer the possibility to verify already existing theories by finding known relations in the decisions of the algorithm. Through the nonlinear combination of features in the tree, new relationships can be found. This combination can challenge existing knowledge if certain features are not used for the decision [13, 14]. The ability of certain algorithms to uncover nonlinear relationships can also generate new knowledge.

In order to demonstrate the aforementioned merits, a C5.0 algorithm was trained to correctly classify four classes of cardiac rhythms. We used a very good and large ECG dataset which is publicly available [15]. The smallest possible number of features was used to achieve very good accuracy. Additionally, the structure of the algorithm was strictly limited. Thus, nonlinear relationships can be extracted from large amounts of data in an understandable way. The aim of this work is to create a clear diagnostic algorithm by evaluating the data of more than 10,000 subjects. The structural relationships can thus be presented and interpreted, which shows the advantages of white-box ML approaches. To put the results into context, the outcomes of other ML approaches are also included. A comparison is made with a generalized linear model (GLM), a multinomial logistic regression model (Logit), and with other white-box and black-box ML approaches.

## Materials and methods

### Dataset

The dataset used for our study originates from Chapman University and Shaoxing People’s Hospital and contains 12-lead ECG data from 10,646 patients recorded in a hospital setting [15]. It consists of 83% patients with cardiac arrhythmias with at least one abnormality and 17% patients with normal sinus rhythm. The ECG segment length for each patient is 10 seconds, and the sampling rate is 500 Hz. Of the total of 10,646 patients, 5,956 are male (55.95%) and 4,690 are female (44.05%). The average age is 51.19 years, with a standard deviation of 18.03 years. The youngest patient is 4 years old, and the oldest is 98 years. The recorded data were labeled by two certified physicians; if they contradicted each other, a final decision was made by a senior physician. A distinction was made between 11 different rhythms, but since a few of the rhythms are rare, some rhythms were combined according to the recommendation of Zheng et al. [15] and the guidelines [16–18], leaving 4 classes at the end. Table 1 shows how the classes were combined. The division into the four classes was performed because of the similar effects of the diseases on the heartbeat. Thus, atrial fibrillation and atrial flutter exhibit similar effects on the ECG and were grouped together in class 0. Atrial flutter often occurs in patients before atrial fibrillation [16]. Many patients with atrial flutter develop atrial fibrillation. In addition, the two arrhythmias may also coexist [17]. Class 1 arrhythmias are a heterogeneous group of tachycardias, or rapid heartbeat, and are therefore combined. Another reason to combine patients with tachycardias is the low number of occurrences of some subtypes.

**Table 1:** The 11 different rhythms and how they are grouped into 4 classes.

Rhythm name	*n*	Class	Total size	male/female	Age
Atrial Fibrillation	1,780	Class 0	2,225	1,298/927	72.9 ±11.68
Atrial Flutter	445				
Supraventricular Tachycardia	587	Class 1	2,307	1,152/1,155	55.44 ±20.49
Atrial Tachycardia	121				
Sinus Atrium to Atrial Wandering Rhythm	7				
Sinus Tachycardia	1,568				
Atrioventricular Node Reentrant Tachycardia	16				
Atrioventricular Reentrant Tachycardia	8				
Sinus Bradycardia	3,889	Class 2	3,889	2,481/1,408	58.34 ±13.95
Sinus Rhythm	1,826	Class 3	2,225	1,025/1,200	50.83 ±19.25
Sinus Irregularity	399				

Class 2 consists of only sinus bradycardia, which describes a slow heartbeat. Consequently, there are no malignant bradyarrhythmias in this class. Sinus irregularity and normal sinus rhythm have been combined in class 3. Sinus irregularities are mostly naturally occurring and benign rhythms, which also occur in a healthy state, especially in young people [19, 20]. By combining the sinus rhythm and sinus irregularity, they can be better distinguished from tachycardias [15]. Besides the tree with four classes, an additional tree was trained, which should distinguish six classes. This tree shows the robustness of the C5.0 with a different database. An interpretation can be found in the S1 File.

### Data preprocessing

In this work, denoised data from Zheng et al. [15] were used. Noise can be generated by power line interference, electrode contact noise, motion artifacts, muscle contraction, baseline wandering and random noise. In order to remove these unwanted influences, several steps were taken to clean the data [15]. First, a Butterworth low-pass filter with a passband of 50 Hz and a stopband of 60 Hz was applied. The parameters were chosen because an ECG lies in the frequency range between 0.5 Hz and 50 Hz, and therefore the signal above 50 Hz is canceled by the Butterworth low pass filter. A local polynomial regression smoother was then used to eliminate baseline wandering. Finally, the non-local means technique was performed to remove the remaining noise.

### Feature selection

A total of 24 features were calculated from the denoised signal. Thirteen of them have already been listed by Zheng et al. [15], and 11 more have been additionally determined. The features are exclusively time-based features, most of which have already been used in many previous studies [21–26]. When selecting features, special care was taken to ensure that features are selected that can reflect the characteristic differences between the four classes. The features used here were the only ones that were tested in the algorithm development process. All features are listed in Table 2. Further features used in research are summarized in S2 File.

**Table 2:** All 24 features described, and the previous works in which they were used.

Feature (Unit)	Description
RMSSD (ms)	Root mean square of Successive Differences; see [21, 22, 27]
HRV Mean (ms)	Mean value of RR-Intervals; see [21, 23]
RR-Interval variation (%)	Standard deviation divided by mean of RR-Intervals; see [24, 25]
Minimum (ms)	Shortest RR-Interval
Maximum (ms)	Longest RR-Interval
Difference (ms)	Longest RR-Interval—Shortest RR-Interval
Mean (mV)	Mean of the ECG signal
Skewness	Skewness of the ECG signal; see [26]
Kurtosis	Kurtosis of the ECG signal; see [26]
SDNN (ms)	Standard deviation of normal RR-Intervals; see [22]
Sex*	Gender of the subject
Age* (Years)	Age of the subject
Ventricular rate* (BPM)	Calculated through time between R-peaks
Atrial rate* (BPM)	Calculated through time between P-waves
QRS Duration* (ms)	Duration of QRS-Complex; see [23]
QT Interval* (ms)	Duration of QT-Interval; see [28]
QT Corrected* (ms)	Duration of corrected QT-Interval; see [29]
R Axis Deviation* (degree)	Right axis deviation in the direction of depolarization
T Axis Deviation* (degree)	T-Wave Axis Deviation indicating abnormal repolarization [29]
QRS Count*	Number of QRS-Complexes
Q Onset* (samples)	Onset of Q-Wave; see [28]
Q Offset* (samples)	Offset of Q-Wave
T Offset* (samples)	Offset of T-Wave; see [28]

Features marked with * originate from Zheng et al. [15]

### Data partitioning

The data were split into two datasets: 80% of the patients were used to train the model, and 20% of the patients were used to test the model and evaluate performance. Since the ECG data involve 10 seconds for each subject, the complete length was used. Therefore, the datasets of the individual subjects were not split, and there are no subjects in the test set who are in the training set. This means that an inter-patient division was conducted. The division of the data into the two groups was performed randomly.

### White-box ML approach

We have chosen the C5.0 algorithm as a classification model for the 4 classes because of its good interpretability and structure-revealing characteristics [9, 30], which are particularly suitable for this work. It is also a widely used and reliable method that is commonly used in medical applications [31, 32].

The tree is built based on the training data and can then be tested using the test data. To build the tree, information gain is calculated for each available feature, and then a tree is formed based on this value. Information gain is determined for each feature by calculating how much information would be gathered by a split using this feature. The feature with the highest information gain becomes the root node of the tree. The tree is then generated based on this principle until no further splitting is possible. The splits are then evaluated, and splits that do not contribute significantly to the performance of the model are removed. This step of pruning must be emphasized in this work. Since the goal is to obtain rules that are easily understandable, it was specified that at least 40 cases (n = 40) must occur for a split to be performed. To build the C5.0 model, we used the C50 package in R.

### Performance metrics and evaluation

To achieve the goal of this work, the tree should be as interpretable as possible. Nevertheless, it is important to create a tree that performs well, so that the rules formed and the insights gained from them are valid. Therefore, we use common and standard performance metrics to evaluate the tree. The selected criteria are *sensitivity*, *specificity*, *positive predictive value*, *negative predictive value* and *balanced accuracy*. The S3 File contains the formulas for calculating the performance metrics.

## Results

The C5.0 model was trained using training data and 10-fold cross-validation to avoid overfitting. The number of trials was set to 1, so that it is possible to visualize the tree. No winnowing was used, and the minimum number of cases to include a split was set to 40. The decision against winnowing was made because a higher accuracy was achieved in this manner. The minimum number of cases was set to 40 to ensure the interpretability of the tree. Table 3 shows the confusion matrix of the decision tree achieved when testing with the testing set.

**Table 3:** Confusion matrix of C5.0 model using the test dataset.

**Prediction**	**Reference**				
		**0**	**1**	**2**	**3**
	**0**	**369**	34	10	15
	**1**	42	**423**	0	2
	**2**	6	0	**771**	0
	**3**	23	1	1	**419**

The confusion matrix shows that a total of 1,982 subjects of the test dataset were classified correctly, and 134 were classified incorrectly. There were a total of three cases that have never been misclassified. Class 1 subjects (tachycardias) have never been predicted as class 2 (sinus bradycardia), and class 2 subjects have never been predicted as class 1. Furthermore, class 3 subjects (sinus rhythm) have never been predicted as class 2.

The performance metrics for each of the individual classes are shown in Table 4. The balanced accuracy over the entire testing set was 95.35%. Furthermore, the robustness of the algorithm was tested. In one test, subjects under 18 years of age were excluded, since their ECGs often differ from those of adults. In a further test, subjects with atrioventricular node reentrant tachycardia and atrioventricular reentrant tachycardia were removed from the dataset, as they constitute only a small number of subjects. The tests led to balanced accuracies of 95.2% and 94.99%, respectively. Thus, the robustness of the algorithm could be verified.

**Table 4:** Performance metrics of C5.0 model using the test dataset.

Performance Metric	Class 0	Class 1	Class 2	Class 3
**Sensitivity**	0.8386	0.9236	0.9859	0.9610
**Specificity**	0.9648	0.9735	0.9955	0.9851
**Positive Predictive Value**	0.8621	0.9058	0.9923	0.9437
**Negative Predictive Value**	0.9579	0.9788	0.9918	0.9898
**Prevalence**	0.2079	0.2164	0.3696	0.2060
**Balanced Accuracy**	0.9017	0.9485	0.9907	0.9731

**Fig 3:**
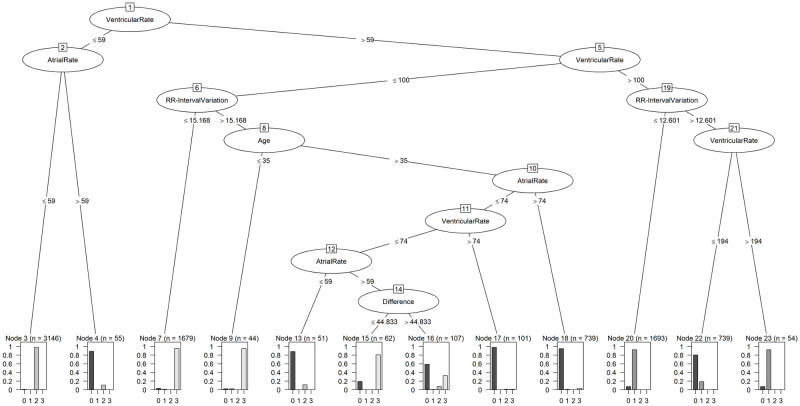
Decision tree from C5.0 model.

The performance metrics show that class 2, with a balanced accuracy of 99.07%, was the best predicted class. Class 3 and class 1 follow with values of 97.31% and 94.85%, respectively, while class 0 (atrial fibrillation and atrial flutter) was the least reliably detected, at 90.17%. In particular, the Negative Predictive Value and the Specificity are very high, which shows that the tree effectively detects that a person is not affected by this rhythm disorder. The resulting tree is shown in Fig 3.

The tree is made up of 7 levels and 11 nodes. The feature usage of the tree is shown in Table 5. The root node of the tree forms the ventricular rate, which therefore represents the highest information gain of all features and is used to distinguish subjects in 100.00% of the cases. With a value of 62.13%, the RR-Interval variation is the feature used for the second-most cases and can therefore be found high up in the tree. The atrial rate is also frequently used, with a usage value of 50.28%. The age and the difference between the maximum and minimum RR-Interval are the least used features of this tree, with values of 13.00% and 2.40%, respectively. In total, the tree uses 5 of the 24 features available to achieve the balanced accuracy of 95.35%. The ventricular rate and the atrial rate are reused after their initial use after several nodes.

**Table 5:** Feature usage of C5.0 model using the test dataset.

Feature	Usage
**Ventricular rate**	100.00%
**RR-Interval variation**	62.13%
**Atrial rate**	50.28%
**Age**	13.00%
**Difference**	2.40%

To better illustrate the advantages of the C5.0, and to frame the result in the context of other ML algorithms, we have trained additional algorithms. For this purpose, extremely high performance algorithms, which are common in the medical context, were selected [33–36]. These results are shown in Table 6. A GLM and Logit-Model were trained as simple models to represent a baseline. These models achieved balanced accuracy of 70.02% and 94.51%, respectively. In order to use other white-box ML approaches [37], a k-Nearest Neighbor (k-NN) and a Naive Bayes Classifier were trained. These achieved 93.21% and 91.66% balanced accuracy. As part of the black-box ML algorithms, Random Forest and eXtreme Gradient Boosting Classifier were chosen. The balanced accuracy values were 96.00% and 95.92%. The low standard deviation further underpins the algorithms’ stability.

**Table 6:** Performance metrics of C5.0 model using the test dataset.

Performance Metric	C5.0	GLM	Logit	k-NN	NB	RF	XGB
**Sensitivity**	0.9273 (± 0.0086)	0.5484 (± 0.0143)	0.9152 (± 0.0094)	0.8954 (± 0.0092)	0.8721 (± 0.0106)	0.9374 (± 0.0062)	0.9363 (± 0.0083)
**Specificity**	0.9797 (± 0.0025)	0.8521 (± 0.0040)	0.9750 (± 0.0026)	0.9687 (± 0.0027)	0.9611 (± 0.0029)	0.9824 (± 0.0016)	0.9820 (± 0.0023)
**Positive Predictive Value**	0.9260 (± 0.0084)	0.5187 (± 0.0123)	0.9105 (± 0.0083)	0.8856 (± 0.0096)	0.8609 (± 0.0101)	0.9373 (± 0.0056)	0.9361 (± 0.0076)
**Negative Predictive Value**	0.9794 (± 0.0025)	0.8426 (± 0.0029)	0.9743 (± 0.0024)	0.9667 (± 0.0029)	0.9593 (± 0.0030)	0.9824 (± 0.0015)	0.9819 (± 0.0021)
**Balanced Accuracy**	0.9535 (± 0.0050)	0.7002 (± 0.0075)	0.9451 (± 0.0077)	0.9321 (± 0.0087)	0.9166 (± 0.0235)	0.9600 (± 0.0067)	0.9592 (± 0.0106)

Abbreviations: GLM = Generalized Linear Model, Logit = Multinomial Logistic Regression, k-NN = k-Nearest Neighbor, NB = Naive Bayes Classifier, RF = Random Forest, XGB = eXtreme Gradient Boosting. Performance metrics presented as mean (± standard deviation).

## Discussion

The advantages of a white-box ML approach mentioned at the beginning of this paper will be clarified in the following by interpreting the resulting tree. For this purpose, the revealed structures will be analyzed in detail and framed in the context of already existing knowledge.

The C5.0 had a total of 23 variables to choose from, of which only five were needed to achieve the balanced accuracy of 95.35%: ventricular rate, RR-Interval variation, atrial rate, age and difference. While the individual features themselves are hardly meaningful in everyday clinical practice, they become relevant to correctly identify the four classes when used in the combination shown here. Furthermore, it must be considered that the features alone cannot be interpreted by the physician at first, but they can be extracted from the raw ECG signals and thus offer possible clinical application.

**Fig 4:**
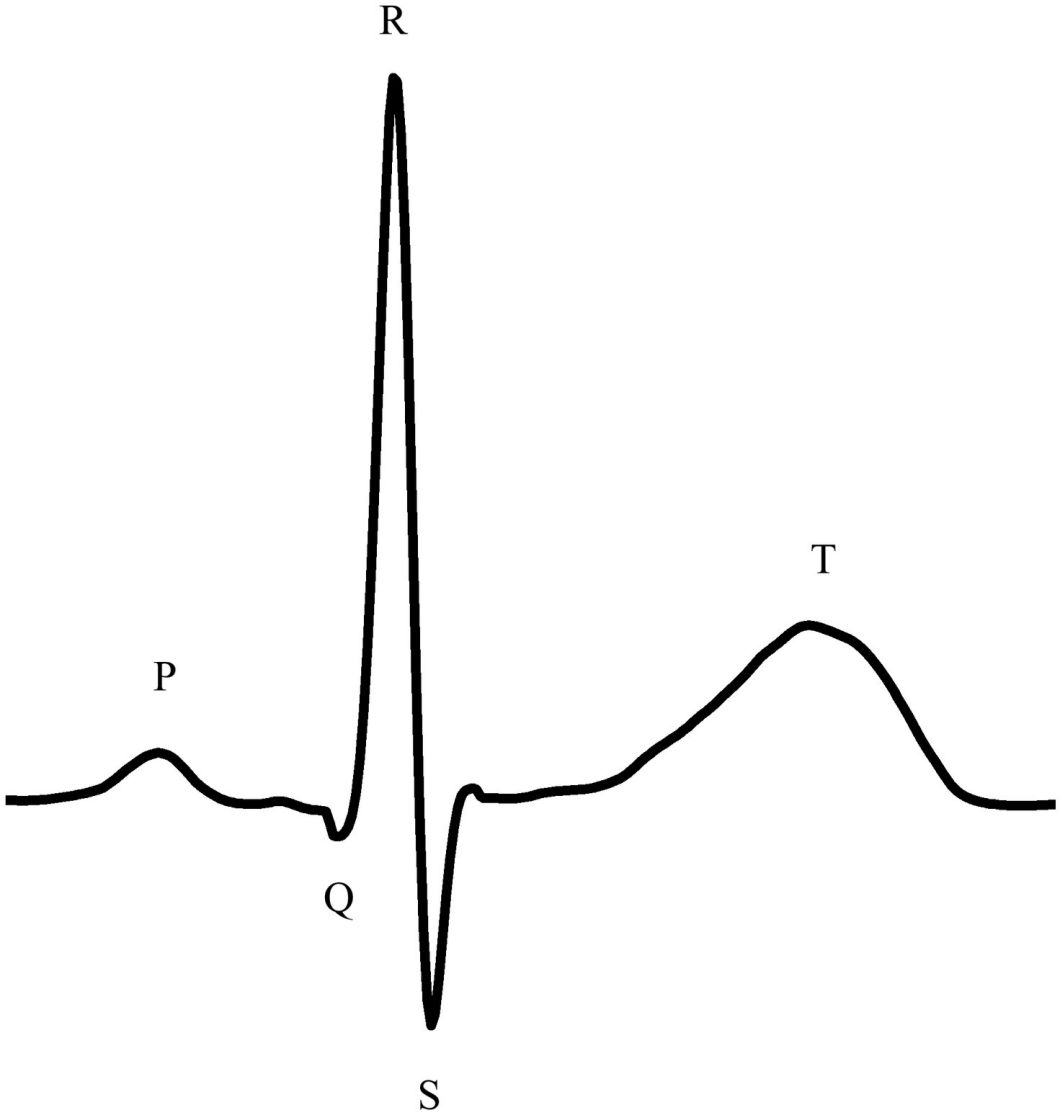
Schematic ECG waveform of a normal cardiac cycle.

Except for the age of the subjects, all features are derived from the ECG signal. The ECG records the excitation state of the heart and its different phases based on the electrical activity, which is shown in Fig 4. The depolarization of the atria can be recognized by the P-wave [38, 39], and the depolarization of the ventricles can be determined by the QRS-Complex [39]. The ST-Segment is an expression of complete depolarization with even distribution of electronegativity. This is followed by the T-wave as an expression of repolarization [40]. Very relevant to this work is the P-wave, which carries the excitation of depolarization of the atria [38, 39]. With this, the atrial rate can be determined. This corresponds to the number of P-waves per minute. Thus, it represents the sum of the excitation states of the atria per minute. The positive R-wave is also important to the algorithm as an expression of the ventricular depolarization. The ventricular rate is the sum of the R-peaks, and thus chamber depolarizations per minute. If no heart disease is present, the ventricular rate and the atrial rate should not vary from each other [41]. The RR-Interval variation is based on the time difference between the R-peaks (RR-Interval). It is calculated by dividing the standard deviation of the RR-Intervals by the average of the RR-Intervals. For the total difference, the largest RR-Interval is subtracted from the smallest RR-Interval. As a last feature, the age of the subjects is used by the algorithm. The advantage of the 5 features is that they are all easy to rapidly calculate.

**Fig 5:**
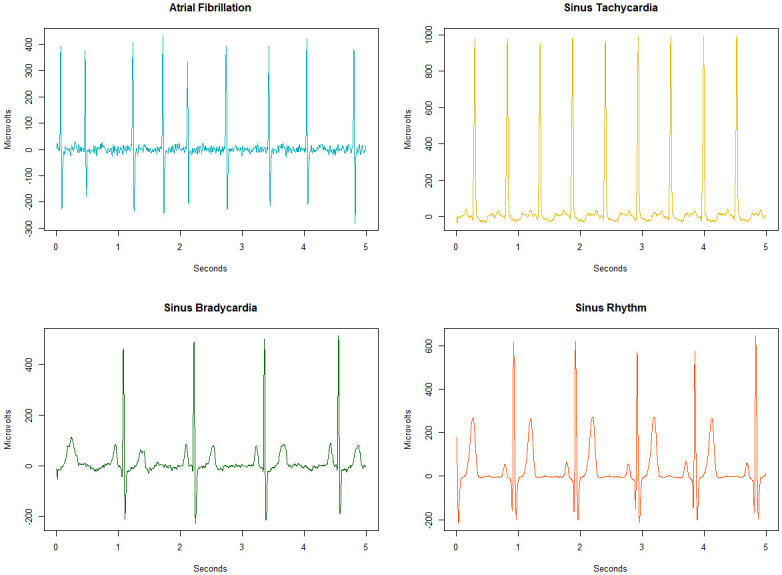
ECG of the 4 classes. Atrial fibrillation as one example of class 0; sinus tachycardia as an example of class 1; class 2 is shown with an exemplary sinus bradycardia signal; a sinus rhythm represents class 3.

Class 0, atrial fibrillation and atrial flutter, is often characterized by rapid irregular atrial activation. As shown in Fig 5 on the upper left side, instead of the P-Wave, an oscillating signal occurs around the baseline. With this increased activity, the atrial rate also increases. While atrial rate and ventricular rate are the same in a healthy heart, there is a difference between them in class 0; due to the increased activity, the atrial rate is potentially higher than the ventricular rate [41]. Class 0 can also lead to significantly increased heartbeat [16, 42]. A ventricular rate of 100 BPM—180 BPM, and sometimes even greater, may occur [16, 41]. Examining class 0 in Fig 4, the oscillation between T- and R-Waves is very clear. However, it should be noted that bradycardia in connection with atrial fibrillation is also not uncommon [43, 44].

Class 1, represented by sinus tachycardia on the upper right side in Fig 5, summarizes six diseases with increased heart rate. While a normal heart rate is between 60 BPM—100 BPM, class 1 diseases lead to heartbeats of over 100 BPM [45]. The healthy heartbeat begins with an electrical impulse from the sinus node [43]. In class 1 diseases, an electrical impulse fires outside the sinus node [46, 47] and thus leads to a significantly increased heartbeat, shortened RR-Intervals and increased ventricular rate. This increased heartbeat causes the heart chambers between the heartbeats to fill incompletely with blood [48]. In Fig 5, the significantly shortened RR-Intervals in class 1 can be observed.

Class 2 includes subjects with sinus bradycardia, meaning a very low heart rate. One example can be seen in Fig 5 on the lower left side. Sinus bradycardia is defined as a heart rate below 60 BPM. In case of an intrinsic reason, this is due to the lack of pulse generation or conduction at the sinoatrial node [49]. However, extrinsic reasons such as autonomously mediated syndromes or neurological disorders may also be responsible [43]. Therapy against bradycardia can be performed using drugs. Sinus bradycardia has various symptoms such as shortness of breath, dizziness or fatigue [49].

Class 3 includes subjects with regular sinus rhythm and subjects with irregularities in sinus rhythm that are not due to class 0, 1 or 2 diseases. Comparing the distances of the RR-Intervals of class 3 (sinus rhythm) and class 2 (sinus bradycardia) in Fig 5, significantly larger distances for class 2 can be seen.

Examining the tree structure of the C5.0, class 0 can be identified with 90.17% success. The ventricular rate is always required, and either the atrial rate in node 2, the RR-Interval variation in node 19 or a combination of all features is used in the other nodes. Of the final nodes, there are 6 that mainly indicate class 0. To distinguish between class 0 and class 1, the algorithm uses the RR-Interval variation. Due to the variation in the ventricular rate in class 0, there is a higher signal variance [50], which is reflected in node 19. In a further split, the algorithm differentiates using the ventricular rate: if it exceeds 194 BPM, class 1 is present, and otherwise class 0 is present.

Class 2 is characterized by a heartbeat of less than 60 BPM. Our algorithm detects class 2 with a balanced accuracy of 99.07% using the ventricular rate and the atrial rate at nodes 1 and 2. If both are below 59 BPM and the atrial rate is not higher than the ventricular rate, class 2 disease is present. If the atrial rate is higher than the ventricular rate, the algorithm classifies node 2 as class 0, and the tree represents the characteristics of atrial fibrillation or atrial flutter.

Class 3 can be recognized by the tree with 97.31% success. It is primarily compared with class 0 in the middle part of the tree. With nodes 1 and 5, the tree narrows down the subset of subjects to those with ventricular rate between 59 BPM and 100 BPM. To distinguish the first subset of subjects in class 3, the RR-Interval variation is used. Patients with atrial fibrillation have greater variation in their heartbeats than class 3 subjects [43, 51]. Accordingly, subjects with RR-Interval variation below 15.168 are assigned to class 3. In order to make further distinctions, age is used as the next criterion. Subjects younger than 35 or aged 35 are also assigned to class 3. This is due to the fact that the variation in heartbeat decreases with age, and a higher RR-Interval variation is therefore not unusual for younger people [52]. Subjects with an atrial and ventricular rate of over 74 are then also assigned to class 0 in nodes 10 and 11. Node 12 again uses the atrial rate for class 0, which is due to the fact that the P-wave is absent, which can occur in atrial fibrillation [53]. In node 14, the difference between the RR-Intervals is used: if it is greater than 44.833 ms, then mostly class 0 is involved, and if it is smaller, class 3 is predominant.

**Fig 6:**
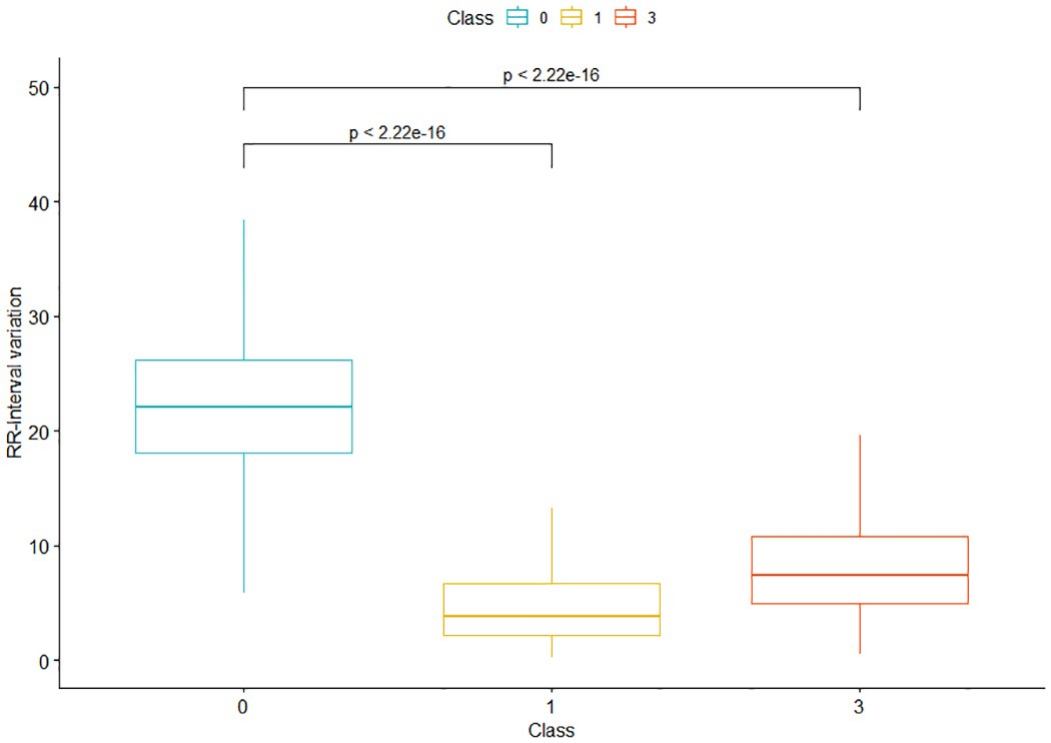
Boxplot for the RR-Interval variation of classes 0, 1 and 3.

As the second-most important feature of the tree, which explicitly describes the variance in the heartbeat and from which important information can be derived, the RR-Interval variation is now examined in more detail. The RR-Interval variation is used to distinguish class 0 from class 3 and to separate class 0 from class 1 (see Fig 6); it is not used to detect class 2. This is linked to the fact that the variation in heartbeat increases with atrial fibrillation, and that the RR-Interval variation is thus higher in subjects from class 0 than in the other two classes [43, 51]. This is reflected as a statistical effect in a Cohen’s d of 2.26 between classes 0 and 1 and a Cohen’s d of 2.04 between classes 0 and 3. In comparison with class 3, the RR-Interval variation was 15.168 in our tree, which matches the findings of van den Berg et al. [54], for instance, who calculated the RR-Interval variation in their work: the result was also below 15.168 in healthy subjects and above 15.168 in subjects with atrial fibrillation. Regarding the variability of the heartbeat, there are findings that it is reduced in subjects with tachycardia [55]. In conjunction with the increased variation in atrial fibrillation, this fits with the split in node 19, which distinguishes between class 0 and class 1 with an RR-Interval variation of 12.601.

This result highlights the relevance of the RR-Interval variation. In combination with the other features used, the basis for the tree’s decision can be clearly understood. As initially pointed out, the white-box ML approach has three advantages: previously known knowledge can be verified or challenged, and new knowledge can be generated. These are also reflected in the structure of the tree. The used features are already known in this context; the combination of the features provides new insights. Monitoring the features may also have implications for understanding the mechanisms of therapy. How the therapy influences the pathophysiology of the disease, and whether it is beneficial, can also be understood.

To interpret the result of the C5.0 in the context of other ML models, further algorithms were selected and trained for classification using the same features. As the results of the standard deviation in Table 6 show, robust models were trained. Stability is of high relevance for ML models, especially for white-box ML models, where reliable information is to be obtained directly from the models [56]. As a baseline model, a GLM was trained to correctly determine the four classes. A balanced accuracy of 70.02% was achieved. The sensitivity, which is the identification of a subject as belonging to a specific class, was only 54.84%. Here, the C5.0 achieved a correct classification of 92.73%. As another simple model, a Logit model was trained, which achieves a balanced accuracy of 94.51%. Compared with the GLM, the Logit model exhibits much better performance. The balanced accuracy is slightly worse than that achieved by the C5.0. Looking at the individual classification results, it is noticeable that the C5.0 recognizes class 1, class 2 and class 3 more accurately (for all results of the Logit model see S4 File).

**Fig 7:**
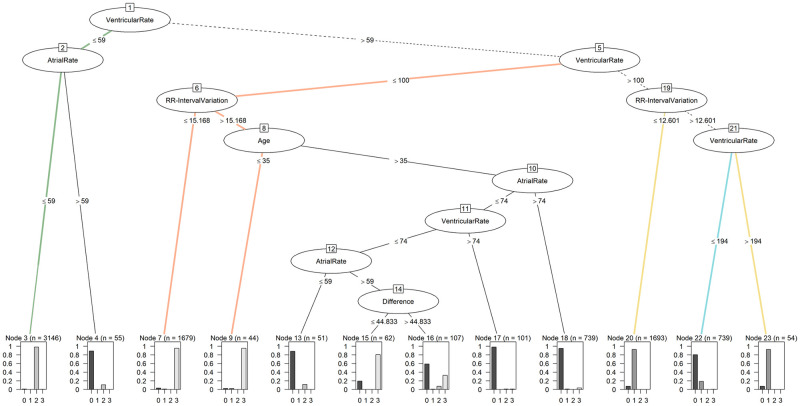
Decision paths of the C5.0 model. Paths to class 0 are shown in cyan, to class 1 in yellow, to class 2 in green and to class 3 in orange. Paths for multiple classes are shown in black dashed lines.

Compared to the other models, the C5.0 can also provide valuable implications for the scientific context. Using the information provided by the algorithm, existing knowledge can be verified and challenged. Furthermore, new insights can be extracted. For this purpose, the decision thresholds were manually analyzed and compared with existing knowledge in cardiology. Further features discovered by the analysis are described in S2 File. The findings of the analysis are summarized in Table 7. The different decision paths are also shown graphically in Fig 7.

**Table 7:** Knowledge verified, challenged and uncovered by the C5.0.

Feature/Feature combination	Class	Publication	Status
Differing atrial rate and ventricular rate	0	[16]	verified
RR-Interval variation	0	[41]	verified
Ventricular rate and atrial rate below 60 BPM	2	[43]	verified
HRV Mean	2	[57]	not verified
RMSSD	0	[27]	not verified
Nonlinear combination of ventricular rate, RR-Interval variation and age	0 and 3	-	new knowledge
Nonlinear combination of ventricular rate and RR-Interval variation	0 and 1	-	new knowledge

Class 0 = Atrial fibrillation and atrial flutter, Class 1 = Tachycardia, Class 2 = Sinus bradycardia, Class 3 = Sinus irregularity and sinus rhythm.

Looking at atrial fibrillation and atrial flutter (class 0; decision path shown in Fig 7 along the black dashed and cyan route), one of the characteristics for detection is that the atrial rate and the ventricular rate are differing. While the atrial rate for atrial fibrillation can reach values of 350 BPM—600 BPM, the ventricular rate lies mostly only at 100 BPM—180 BPM. In addition, there is an irregular ventricular rhythm [41]. The decision of the C5.0 is also based on these principles. Therefore, C5.0 recognizes class 0 on the basis of differing atrial rate and ventricular rate. As a further feature, the tree uses the RR-Interval variation, which reflects the irregularities in the ventricular rhythm. Together with the fact that class 0 is well detected, this can be seen as a further confirmation of the suitability of the rules used in practice and science [16, 17].

While bradycardia (shown in green in Fig 7) can also be detected by heart rate variability [57], this feature is not included in the C5.0 for this setting. It only uses the ventricular rate and the atrial rate for class 2. If these two rates are below 60 BPM, bradycardia is present [43]. Accordingly, these two characteristics are more descriptive for correct classification than heart rate variability. Not using given features by the algorithm also allows implications about their information values. The information value is accordingly lower than the information value of the features used by the tree.

This can also be seen in the detection of class 0. As mentioned above, an irregularity in the ventricular rhythm is characteristic. Here the C5.0 can choose between the features RMSSD, RR-Interval variation, SDNN and HRV mean, which describe these irregularities. While in other studies the focus is, for example, on RMSSD [27], the C5.0 decides to use the RR-Interval variation.

Atrial fibrillation, atrial flutter and tachycardia (class 0 and class 1; shown in yellow and cyan) can have similar characteristics. Here the C5.0 reveals nonlinear relationships that can be used for differentiation. If the ventricular rate is above 100 BPM and the RR-Interval variation is below 12.601, class 1 is identified, and if the RR-Interval variation is above 12.601, it is not possible to identify one of the classes with certainty. For this purpose, the ventricular rate is used once again. If the ventricular rate is above 194 BPM, the C5.0 suggests class 1, whereas a ventricular rate below 194 BPM indicates class 0.

The tree uses another nonlinear combination (shown in orange) to distinguish between class 0 and class 3 (Sinus irregularity and sinus rhythm). First, it is determined whether the ventricular rate is between 60 BPM and 100 BPM. Then the RR-Interval variation is considered. If it is below 15.168, it is class 3; if not, a further distinction must be made based on age. If this is less than 35 years, it is class 3. If it is greater than 35 years, it is mostly class 0. Especially these nonlinear correlations, in particular, can lead to relevant new findings.

The use of the features of the C5.0 can strengthen or question the relevance of existing knowledge. By combining the features, the resulting comprehensible decision structure can reveal nonlinear relationships, which in their interplay allow new implications and hypotheses. These are scientific, verifiable statements according to Popper’s understanding. This possibility distinguishes the C5.0 significantly from other white-box ML approaches. While white-box ML approaches reveal more information than black-box ML approaches, they differ internally in the degree of usability of the revealed structures. In particular regarding the exploration of nonlinear relationships, the C5.0 with its decision tree basis offers considerable advantages compared to, for instance, the Logit model. Such findings cannot be made based on the results of the Logit model. This only outputs coefficients with information about their absolute relevance in the model. Explicit statements about the decision thresholds cannot be made. Consequently, there are levels of interpretability between the different white-box ML approaches. As a result of the exposed and comprehensible knowledge, the discourse in the domain can be enriched and the trust of domain experts can be increased [7].

**Fig 8:**
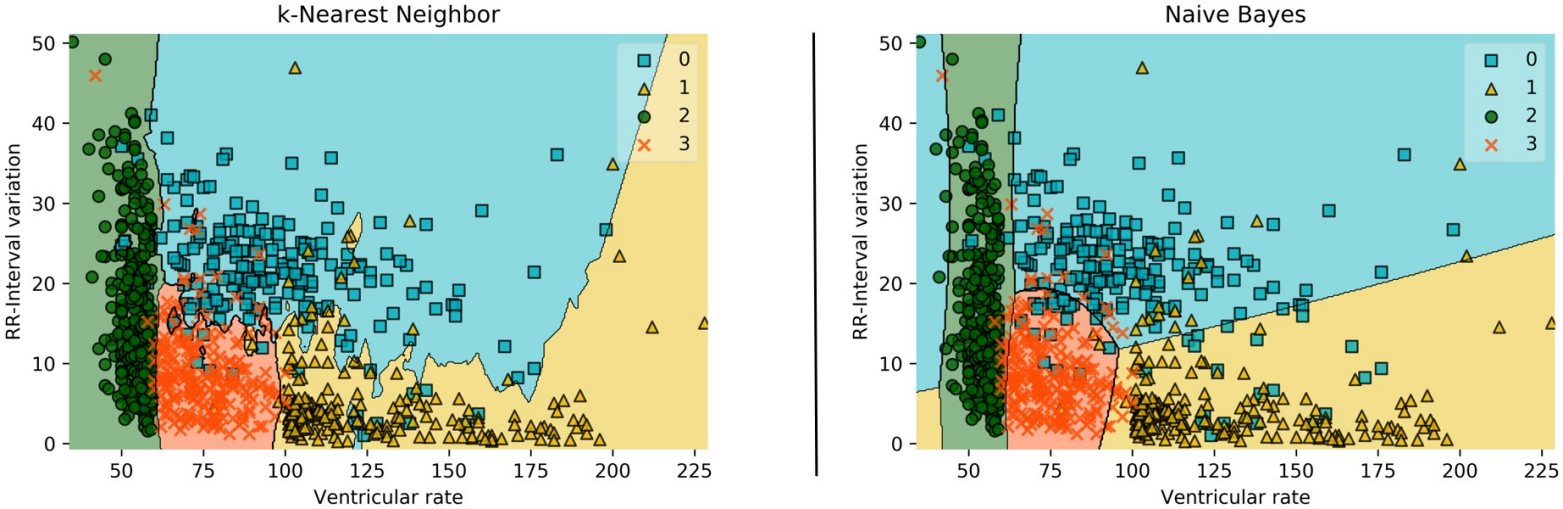
Visualization of the two best features of k-NN and Naive Bayes Classifier. For clarity, only 1,000 randomly selected patients were included in the visualization.

In addition, two further white-box ML approaches were used to allow comparison. The k-NN algorithm achieved a balanced accuracy of 93.21%, and the Naive Bayes classifier achieved 91.66%. Besides the fact that the algorithms performed slightly worse than the C5.0, C5.0 offers a more common structure for the interpretation of the results [31, 32]. This can be seen in Fig 8. Comparing this representation with the visualization in Fig 3, it is noticeable that the tree offers the possibility to completely comprehend all features in the overall structure. In the visualization of the decision boundaries of k-NN and Naive Bayes Classifier, only the two best features, ventricular rate and RR-Interval variation, could be displayed. The inclusion of an additional feature would make the representation more complex and would be difficult to interpret. Considering that the C5.0 uses a total of five features, the advantage of its tree-based structure for interpretability becomes even clearer.

Black-box ML approaches were also used to compare their performance with the C5.0. For this purpose, the Random Forest Algorithm and eXtreme Gradient Boosting Classifier were used. Random Forest achieved a balanced accuracy of 96%, and that of the eXtreme Gradient Boosting Classifier was 95.92%. Thus, the two classifiers based on deep decision tree structures were able to deliver slightly better results than the C5.0.

As the accuracies of the presented black-box ML approaches show, these approaches can achieve excellent results. This can also be observed in other application domains [1–4]. However, as can also be seen in Fig 1, they are difficult or impossible to understand [5]. Since the black-box approaches have increasingly faced criticism, attempts have been made to explain their structures. For this purpose, a second model is being created to explain the black-box model. However, these explanations are often unreliable, misleading and therefore problematic [6]. Table 8 shows an overview of the white-box and black-box ML algorithms used in this paper, taking structural insight and accuracy into account. It can be seen that the white-box ML approaches k-NN and Naive Bayes are structure revealing, but have lower accuracy. Examining Fig 8, it can also be determined that the revealing structure, at least in a single diagram, only provides a limited overall impression of the relationships. In contrast, black-box ML algorithms have a high degree of accuracy, but their structures may only be visualized with great difficulty, if at all. With the C5.0 algorithm used here, complex relationships between the variables can be displayed and comparable accuracies can be achieved.

**Table 8:** Comparison of white-box and black-box ML models in terms of their structure revelation and accuracy of prediction.

Classifier	Structure revelation	Prediction accuracy
**k-Nearest Neighbor**	+	-
**Naive Bayes Classifier**	+	-
**Random Forest**	-	+
**eXtreme Gradient Boosting**	-	+
**C5.0**	+	+

It is frequently argued that the error rate in diagnosing disease with black-box ML algorithms is lower than that of practicing physicians, which is why the algorithms should be used in medical clinics. Considering the value of individual health, this proposal is questionable, at least from an ethical point of view, because an algorithm whose operating principles cannot or can only barely be grasped would therefore detect diseases and recommend treatment methods. In the event of errors, it would be difficult in this case to understand how a decision was reached. By means of structure-revealing white-box ML approaches using algorithms such as C5.0, how results are obtained can be clearly reconstructed. Clear tree structures can thus be used as a tool to extract nonlinear relationships and thus to extract essential information from large amounts of data. The structures are therefore ideally suited for use in medical applications where this is crucial. The extracted criteria for classification can then be implemented by physicians in everyday clinical practice. In this work, great importance was therefore attached to using features that are easy to calculate. The tree structure was also greatly restricted. Nevertheless, an excellent result of 95.35% has been achieved. This supports the view that there is not necessarily a compromise with respect to accuracy and interpretability [6].

The detection of cardiovascular diseases is extremely complex in everyday clinical practice [1]. The white-box tree structure developed here could be used by physicians to support them in finding a diagnosis.

## Limitations

In this work, a decision tree was selected as algorithm due to its interpretability. However, it must be mentioned that there are also limitations of this method. A changed data basis can lead to instability in the model. Furthermore, a decision tree cannot efficiently represent linear relationships. As far as the grouping of classes is concerned, the individual diseases were combined into four classes according to the guidelines [16–18].

## Conclusion

The white-box ML approach presented here uses a C5.0 model to classify cardiovascular rhythms based on features extracted from ECG data. All relevant features are learned by the tree in order to distinguish between 4 classes of cardiac rhythms with very high accuracy. The structure revealing characteristics of the tree allow discovery of nonlinear relationships which may be important for clinical practice and for a better understanding of diseases. Here, it is necessary to emphasize that the combination of the features made by the tree provides an especially important benefit. Thus, the majority of the test persons of the 4 classes can be classified by a combination of the three most important features. Furthermore, the features used are easy to derive from the ECG and may be used by physicians for diagnosis. In conclusion, it can be said that structure-revealing white-box ML approaches provide excellent added value in the detection of diseases.

## Supporting information

S1 File C5.0 performance with six classes. (DOCX)

S2 File Features in literature. (DOCX)

S3 File Performance metrics. (DOCX)

S4 File Performance comparison Logit model. (DOCX)
