# Supplementary material for: Demonstration of the potential of white-box machine learning approaches to gain insights from cardiovascular disease electrocardiograms
Source: PLoS One. 2020 Dec 17;15(12):e0243615. doi: 10.1371/journal.pone.0243615 (PMC7746264; doi:10.1371/journal.pone.0243615)
Supplement: S1 File — (DOCX) [file pone.0243615.s001.docx]

**C5.0 Performance with six classes.**

For further analysis of the heart rhythms, an evaluation was made for six classes. Class 0 was divided into atrial fibrillation and atrial flutter and class 3 into sinus irregularity and sinus rhythm. The individual accuracies can be seen in the following table.

|  | **Atrial Fibrillation** | **Atrial Flutter** | **Tachycardia** | **Sinus Bradycardia** | **Sinus Irregularity** | **Sinus Rhythm** |
| --- | --- | --- | --- | --- | --- | --- |
| **Balanced Accuracy C5.0** | 0.8697 | 0.7092 | 0.9259 | 0.9887 | 0.7654 | 0.9036 |

**Table 1. Performance of C5.0 for 6 classes.**

As can be seen, atrial fibrillation is classified with 86.97% accuracy and atrial flutter with 70.92% accuracy. Tachycardia has an accuracy of 92.59% and Sinus Bradycardia has an accuracy of 98.87%. For sinus irregularity an accuracy of 76.54% and for sinus rhythm an accuracy of 90.36% was achieved. The balanced accuracy over all classes is 82.9%.

While the original tree was trained with largely balanced classes, the new split has class imbalances, as there are only 445 cases for atrial flutter and 399 cases for sinus irregularity. This has the consequence that a minimum number of cases per split of n = 40 cannot be maintained but had to be reduced to n = 20. As a result, the tree is deeper and consequently consists of 11 levels and 40 nodes. It was decided to combine rhythms with similar characteristics to a balanced data set in order to demonstrate the advantages of the C5.0 as a white-box ML approach.

To verify the robustness of the approach chosen here the division into six classes was used to compare the relevant features. These can be seen for both four and six classes in the following tables.

| **C5.0 with 4 classes** | |
| --- | --- |
| **Feature** | **Usage** |
| **Ventricular rate** | 100.00% |
| **RR-Interval variation** | 62.13% |
| **Atrial rate** | 50.28% |
| **Age** | 13.00% |
| **Difference** | 2.40% |

**Table 2. Feature usage of C5.0 for 4 classes.**

| **C5.0 with 6 classes** | |
| --- | --- |
| **Feature** | **Usage** |
| **Ventricular rate** | 100.00% |
| **Atrial rate** | 94.13% |
| **RR-Interval variation** | 60.48% |
| **Age** | 33.11% |
| **Difference** | 29.34% |

**Table 3. Feature usage of C5.0 for 6 classes.**

The five most used features in this case were exactly the same as in the C5.0 for four classes. Only the usage of the features differs from the original distribution. Looking more closely at this usage, it is noticeable that the atrial rate becomes more relevant for six classes. This can be explained by the fact that atrial flutter has an irregularity in the rhythm, which is reflected in a difference between atrial rate and ventricular rate. Atrial flutter can result in an increased atrial rate, which is often 2:1 to the ventricular rate [1]. In atrial flutter, atrial contractions of approximately 300 BPM occur due to a re-entry circuit in the right atrium with secondary activation of the left atrium [1]. Atrial flutter is a rhythm that often precedes atrial fibrillation [2, 3, 4]. Many patients with atrial flutter develop atrial fibrillation in the future [3]. Furthermore, both diseases can coexist [2, 3]. With sinus irregularity, the morphology of the P-wave is normal, but the time between P-waves varies relatively strongly. This is a benign rhythm that can sometimes be influenced by respiration in young people [5, 6]. Due to the size of the tree, the features age and difference are on a higher level which means that there are more cases in the splits underneath and therefore the usage percentage is higher.

Considering the different database used to train the two C5.0's, this result speaks for the robustness of the rules underlying the tree. Thus, it can be proven that the features presented in our original tree are highly relevant in this setting even with changing data.

1. Goodacre S. ABC of clinical electrocardiography: Atrial arrhythmias. BMJ. 2002;324(7337):594–597. doi:10.1136/bmj.324.7337.594.

2. Kirchhof P, Benussi S, Kotecha D, Ahlsson A, Atar D, Casadei B, et al. 2016 ESC Guidelines for the management of atrial fibrillation developed in collaboration with EACTS. European Journal of Cardio-Thoracic Surgery. 2016;50(5):e1–e88. doi:10.1093/ejcts/ezw313.

3. January CT, Wann LS, Alpert JS, Calkins H, Cigarroa JE, Cleveland JC, et al. 2014 AHA/ACC/HRS Guideline for the Management of Patients With Atrial Fibrillation. Journal of the American College of Cardiology. 2014;64(21):e1–e76. doi:10.1016/j.jacc.2014.03.022

4. Brembilla-Perrot B, Girerd N, Sellal J, Oliver A, Manenti V, Villemin T et al. Risk of Atrial Fibrillation After Atrial Flutter Ablation: Impact of AF History, Gender, and Antiarrhythmic Drug Medication. Journal of Cardiovascular Electrophysiology. 2014;25(8):813-820.

5. Denver JW, Reed SF, Porges SW. Methodological issues in the quantification of respiratory sinus arrhythmia. Biological Psychology. 2007;74(2):286–294. doi:10.1016/j.biopsycho.2005.09.005.

6. Ben-Tal A, Shamailov SS, Paton JFR. Evaluating the physiological significance of respiratory sinus arrhythmia: looking beyond ventilation-perfusion efficiency. The Journal of Physiology. 2012;590(8):1989–2008. doi:10.1113/jphysiol.2011.222422.
