# Supplementary material for: Demonstration of the potential of white-box machine learning approaches to gain insights from cardiovascular disease electrocardiograms
Source: PLoS One. 2020 Dec 17;15(12):e0243615. doi: 10.1371/journal.pone.0243615 (PMC7746264; doi:10.1371/journal.pone.0243615)
Supplement: S2 File — (DOCX) [file pone.0243615.s002.docx]

**Features for the recognition of the heart rhythms**

| Class | Feature | Characteristic | Reference | |
| --- | --- | --- | --- | --- |
| Atrial Fibrillation | Ventricular rate | Rapid ventricular response (between 90 BPM and 170 BPM) | [1] |  |
|  | Prolonged PR- Interval | Prolonged PR-Interval, greater than 200ms | [2] |  |
|  | P-Index | P-Index > 35, generating a Hazard Rate of 2.7 | [2] |  |
|  | P-wave duration | Short and long P-wave duration | [3] |  |
|  | RMSSD/ Shannon Entropy | Threshold value of 0.115 for RMSSD/mean and 0.55 for Shannon Entropy | [4] |  |
|  | QT Interval | Prolonged QT-Interval is associated with an increased risk of incident Atrial Fibrillation | [5] |  |
|  | RR-Interval, P-wave | Absolutely irregular RR-Intervals and no discernible, distinct P-wave | [6] |  |
| Atrial Flutter | F-wave | Highly regular F-wave at a rate ≤350 BPM | [7] |  |
|  | ECG-Pattern | A characteristic ECG “sawtooth”-pattern is present inleads II, III and/or aVF. | [8] |  |
|  | RR-Interval | Irregularly irregular RR-Intervals are common in Atrial Flutter | [9] |  |
|  | Atrial Rate | An Atrial Rate close to 300 BPM | [10] |  |
| Tachycardia | QRS-Complex | Duration of QRS-Complex of >0.12 seconds and ventricular rate of > 120 BPM for complex tachycardia | [11] | |
|  | HRV | Low HRV is associated with increased risk of ventricular tachycardia | [12] | |
|  | R-wave | Duration of R-wave in V1 or V2 of >30ms for ventricular tachycardia | [13] | |
|  | Q-wave | Any Q-wave in V6 for ventricular tachycardia | [13] | |
|  | QRS-Complex/S-wave | More than 60ms from QRS-Onset to the nadir of the S-wave in V1 or V2 for ventricular tachycardia | [13] | |
|  | QRS-Duration | Duration over 120ms for ventricular tachycardia or supraventricular rhythm with abnormal conduction | [14] | |
| Bradycardia | Heart Rate | Sinus Rhythm with a resting heart rate of 60 beats per minute or less | [15] | |
|  | HRV | Total HRV is increased | [15] | |
|  | RMSSD | An increase in RMSSD | [15] | |
| Sinus Irregularity | RR-Interval variation | RR-Interval shortened during inspiration and prolonged during expiration | [16] | |

**References**

[1] Gutierrez C, Blanchard DG. Diagnosis and Treatment of Atrial Fibrillation. American Family Physician. 2016;94(6):442–452.

[2] Perez MV, Dewey FE, Marcus R, Ashley EA, Al-Ahmad AA, Wang PJ, et al. Electrocardiographic predictors of atrial fibrillation. American Heart Journal. 2009;158(4):622–628. doi:10.1016/j.ahj.2009.0.

[3] Nielsen JB, Kuehl JT, Pietersen A, Graff C, Lind B, Struijk JJ, et al. P-wave duration and the risk of atrial fibrillation: Results from the Copenhagen ECG Study. Heart Rhythm. 2015;12(9):1887–1895. doi:10.1016/j.hrthm.2015.04.0.

[4] McManus DD, Lee J, Maitas O, Esa N, Pidikiti R, Carlucci A, et al. A novel application for the detection of an irregular pulse using an iPhone4S in patients with atrial fibrillation. Heart Rhythm. 2013;10(3):315–319. doi:10.1016/j.hrthm.2012.12.0.

[5] Mandyam MC, Soliman EZ, Alonso A, Dewland TA, Heckbert SR, Vittinghoff E, et al. The QT Interval and Risk of Incident Atrial Fibrillation. Heart Rhythm. 2013;10(10):1562–1568. doi:10.1016/j.hrthm.2013.07.023.

[6] Kirchhof P, Benussi S, Kotecha D, Ahlsson A, Atar D, Casadei B, et al. 2016 ESC Guidelines for the management of atrial fibrillation developed in collaboration with EACTS. European Journal of Cardio-Thoracic Surgery. 2016;50(5):e1–e88. doi:10.1093/ejcts/ezw313.

[7] Granada J, Uribe W, Chyou PH, Maassen K, Vierkant R, Smith PN, et al. Incidence and Predictors of Atrial Flutter in the General Population. Journal of the American College of Cardiology. 2000;36(7):2242–2.

[8] Saoudi N. A classification of atrial flutter and regular atrial tachycardia according to electrophysiological mechanisms and anatomical bases. A Statement from a Joint Expert Group from the Working Group of Arrhythmias of the European Society of Cardiology and the North American Society of Pacing and Electrophysiology. European Heart Journal. 2001;22(14):1162–1182. doi:10.1053/euhj.2001.2658.

[9] Krummen DE, Feld GK, Narayan SM. Diagnostic Accuracy of Irregularly Irregular RR Intervals in Separating Atrial Fibrillation from Atrial Flutter. The American Journal of Cardiology. 2006;98(2):209-214. doi:10.1016/j.amjcard.2006.01.088.

[10] Wells Jr J, MacLean W, James TN, Waldo AL. Characterization of Atrial Flutter. Studies in Man After Open Heart Surgery Using Fixed Atrial Electrodes. Circulation. 1979;60(3):665–673.

[11] Brady WJ, Skiles J. Wide QRS complex tachycardia: ECG differential diagnosis. The American Journal of Emergency Medicine. 1999;17(4):376–381. doi:10.1016/s0735-6757(99)90091-8.

[12] Huikuri HV, Valkama JO, Airaksinen KE, Seppaenen T, Kessler KM, Takkunen JT, et al. Frequency Domain Measures of Heart Rate Variability Before the Onset of Nonsustained and Sustained Ventricular Tachycardia in Patients With Coronary Artery Disease. Circulation. 1993;87(4):1220–1228. doi:10.1161/01.cir.87.4.1220.

[13] Kindwall KE, Brown J, Josephson ME. Electrocardiographic criteria for ventricular tachycardia in wide complex left bundle branch block morphology tachycardias. The American Journal of Cardiology. 1988;61(15):1279–1283. doi:10.1016/0002-9149(88)91169-1.

[14] Page RL, Joglar JA, Caldwell MA, Calkins H, Conti JB, Deal BJ, et al. 2015 ACC/AHA/HRS Guideline for the Management of Adult Patients With Supraventricular Tachycardia. Circulation. 2016;133(14). doi:10.1161/cir.0000000000000311.

[15] McLachlan CS, Ocsan R, Spence I, Hambly B, Matthews S, Wang L, et al. Increased Total Heart Rate Variability and Enhanced Cardiac Vagal Autonomic Activity in Healthy Humans with Sinus Bradycardia. Increased Total Heart Rate Variability and Enhanced Cardiac Vagal Autonomic Activity in Healthy Humans with Sinus Bradycardia. Baylor University Medical Center Proceedings. 2010;23(4):368–370.doi:10.1080/08998280.2010.11928655.

[16] Yasuma F, Hayano J. Respiratory Sinus Arrhythmia. Chest. 2004;125(2):683–690. doi:10.1378/chest.125.2.6.
