# Supplementary material for: Demonstration of the potential of white-box machine learning approaches to gain insights from cardiovascular disease electrocardiograms
Source: PLoS One. 2020 Dec 17;15(12):e0243615. doi: 10.1371/journal.pone.0243615 (PMC7746264; doi:10.1371/journal.pone.0243615)
Supplement: S3 File — (DOCX) [file pone.0243615.s003.docx]

**Performance Metrics and Evaluation.**

Sensitivity = $\frac{True Positive}{True Positive+False Negative}$

Specificity = $\frac{True Negative}{False Positive+True Negative}$

Positive Predictive Value = $\frac{True Positive}{True Positive+False Positive}$

Negative Predictive Value = $\frac{True Negative}{True Negative+False Negative}$

Balanced Accuracy = $\frac{1}{2}( \frac{True Positive}{Positive}+\frac{True Negative}{Negative} )$
