# Supplementary material for: Demonstration of the potential of white-box machine learning approaches to gain insights from cardiovascular disease electrocardiograms
Source: PLoS One. 2020 Dec 17;15(12):e0243615. doi: 10.1371/journal.pone.0243615 (PMC7746264; doi:10.1371/journal.pone.0243615)
Supplement: S4 File — (DOCX) [file pone.0243615.s004.docx]

**Performance comparison Logit model.**

|  | **Class 0**  **(Atrial Fibrillation/**  **Atrial Flutter)** | **Class 1**  **(Tachycardia)** | **Class 2**  **(Sinus Bradycardia)** | **Class 3**  **(Sinus Rhythm/**  **Sinus Irregularity)** |
| --- | --- | --- | --- | --- |
| **Balanced Accuracy C5.0** | 0.9017 | 0.9485 | 0.9907 | 0.9731 |
| **Balanced Accuracy Logit** | 0.9103 | 0.9309 | 0.9766 | 0.9626 |

**Table 1. Comparison of performance of C5.0 and Logit model for 4 classes.**

**Table 2. Feature Importance of Logit model.**

| **Feature** | **Importance** |
| --- | --- |
| **RMSSD** | 11.1878 |
| **HRV Mean** | 9.8495 |
| **SDNN** | 3.9930 |
| **RR-Interval variation** | 1.2151 |
| **Ventricular rate** | 0.9370 |

**Table 3. Coefficients of Logit model.**

| **Class** | **RMSSD** | **HRV Mean** | **SDNN** | **RR-Interval variation** | **Ventricular rate** |
| --- | --- | --- | --- | --- | --- |
| **1** | 3.9848 | -3.6776 | -1.0400 | 0.0324 | 0.0026 |
| **2** | 3.1185 | -2.3669 | -1.9688 | 1.1326 | -0.0434 |
| **3** | 4.0844 | -3.8050 | -0.9842 | -0.0501 | -0.8909 |
